# Application of enhanced recovery after surgery in perioperative management of patients undergoing laparoscopic surgery for benign gynecological conditions

**DOI:** 10.1097/MD.0000000000043161

**Published:** 2025-07-18

**Authors:** Rouzi Nuermanguli, Deng Jing, Yan JiangYing, Hou Yu

**Affiliations:** a Department of Gynecology, Guangyuan Central Hospital, Guangyuan, China.

**Keywords:** clinical outcomes, enhanced recovery after surgery, laparoscopic gynecological surgery, nutritional status, perioperative care, stress response

## Abstract

This study evaluates the effectiveness of enhanced recovery after surgery (ERAS) protocol in laparoscopic gynecological surgery and its impact on perioperative management. A retrospective analysis was conducted on 187 patients who underwent elective laparoscopic gynecological surgery at Guangyuan Central Hospital between November 2022 and December 2023. Of these, 92 patients were enrolled in the ERAS group, and 95 were assigned to the conventional care group. The ERAS protocol included evidence-based interventions such as shortened fasting duration, carbohydrate loading, avoidance of mechanical bowel preparation, and early postoperative mobilization. Primary endpoints included stress response markers (serum cortisol, homeostatic model assessment of insulin resistance), nutritional parameters (prealbumin, albumin, blood urea nitrogen), and clinical outcomes (time to gastrointestinal recovery, length of hospital stay). Secondary endpoints comprised postoperative complications, healthcare costs, and patient satisfaction. The ERAS group showed significantly faster gastrointestinal recovery (first flatus: 15.54 ± 1.73 vs 21.24 ± 3.53 hours, *P* < .001), shorter hospitalization (4.91 ± 0.90 vs 6.29 ± 1.25 days, *P* < .001), and lower healthcare costs (13,960 ± 1967 vs 15,270 ± 2856 yuan, *P* < .001). Postoperative stress response was reduced (cortisol, *P* = .012), and nutritional parameters were better preserved (*P* < .001). Patient satisfaction was higher in the ERAS group (100% vs 96.8%, *P* = .042). The ERAS protocol significantly optimizes perioperative management for patients undergoing laparoscopic gynecological surgery, reducing hospital stay, lowering complication rates, and enhancing patient satisfaction, thus providing robust evidence for clinical implementation. However, further research is needed to explore its long-term effects.

## 1. Introduction

With ongoing advances in life sciences, extensive research on perioperative pathophysiology, and advances in anesthetic techniques, minimally invasive surgery, and perioperative nursing measures, patient outcomes have significantly improved. These collective breakthroughs have given rise to the enhanced recovery after surgery (ERAS) protocol,^[1,2]^ a multidisciplinary approach designed to optimize every phase of the perioperative journey. ERAS aims to optimize all aspects of the perioperative period, enhance patient recovery quality, reduce postoperative complications, shorten hospital stays, and lower healthcare costs.^[3]^ In gynecology, laparoscopic surgery has gradually become the preferred treatment for various gynecological conditions due to its minimally invasive nature and rapid recovery. However, traditional perioperative care models often overemphasize surgical technicalities while overlooking the holistic physiological and psychological needs of patients.^[4,5]^ This approach can lead to higher rates of postoperative complications, prolonged hospital stays, and inefficient utilization of medical resources. Therefore, there is an urgent need for systematic optimization of perioperative management.

The core of the ERAS protocol lies in multidisciplinary collaboration, incorporating multiple aspects of the perioperative period to reduce both physiological and psychological stress responses in patients and accelerate postoperative recovery. The protocol includes preoperative patient education and nutritional support, optimal anesthesia management and minimally invasive techniques during surgery, and postoperative pain management, early mobilization, and appropriate dietary intake.^[6,7]^ Implementing ERAS protocols not only facilitates postoperative recovery but also enables more efficient resource utilization for hospitals.^[8]^ Female patients, due to their unique physiological and psychological characteristics, are often more sensitive to surgical trauma. Thus, the application of the ERAS protocol is closely related to significant improvements in patient outcomes in the gynecological field.^[9]^ Although numerous studies have explored the effects of ERAS in various surgical disciplines, controversies remain regarding optimal timing of early mobilization, appropriate fasting duration, and the necessity of mechanical bowel preparation in benign gynecological laparoscopic surgery. This study aims to comprehensively evaluate the application effects of the ERAS protocol in the perioperative management of patients undergoing laparoscopic gynecological surgery. Unlike previous studies that focused mainly on single aspects of recovery, our research comprehensively evaluates multiple outcomes by comparing multiple outcome measures between traditional perioperative management and the ERAS model, including postoperative complication rates, length of hospital stay, and patient satisfaction.

## 2. Materials and methods

### 2.1. Study design and population

This study was conducted at Guangyuan Central Hospital between November 2022 and December 2023, following approval from the hospital’s Ethics Committee (Approval Number: GYZXLL2023007). All procedures adhered to the principles outlined in the Declaration of Helsinki. The inclusion criteria were as follows: women aged ≥18 years diagnosed with benign gynecological conditions, scheduled for elective laparoscopic surgery under general anesthesia, with good overall health, the ability to tolerate both surgery and anesthesia, and no significant medical or surgical comorbidities or psychiatric history. Patients were excluded if they had stage III or IV endometriosis, gynecologic malignancies, active pelvic inflammatory disease, indications for emergency surgery, gastrointestinal dysfunction, severe systemic diseases, or a history of psychiatric disorders.

### 2.2. Interventions

The ERAS protocol comprised structured preoperative and postoperative interventions. Preoperatively, patients received structured education and psychological counseling, followed by a modified fasting protocol (6 hours for solid food, 2 hours for clear fluids) and oral carbohydrate loading (250 mL of 20% glucose solution) 2 hours before surgery, with no routine mechanical bowel preparation. Postoperative management included early oral hydration (within 6 hours), early mobilization (within 24 hours), gum chewing on postoperative day 1, early removal of urinary catheter (within 12–24 hours), and thromboprophylaxis with compression stockings. The control group received standard perioperative care, including traditional preoperative counseling, 12-hour fasting period, routine mechanical bowel preparation, and standard postoperative care following institutional guidelines.

### 2.3. Outcome measures

Primary outcomes included stress response markers [homeostatic model assessment of insulin resistance (HOMA-IR) and serum cortisol] and nutritional parameters (prealbumin, albumin, and blood urea nitrogen), measured at baseline and 24 hours post-surgery. Secondary outcomes encompassed clinical parameters (time to first flatus and bowel movement, time to oral fluid intake, length of hospital stay, and total healthcare costs), postoperative complications (nausea, vomiting, surgical site infections, deep vein thrombosis, urinary tract infections, and other adverse events), and patient satisfaction. Patient satisfaction was assessed using a validated questionnaire at 30 days post-surgery and categorized as satisfied, fairly satisfied, or dissatisfied.

### 2.4. Statistical analysis

Data analysis was performed using R version 4.2.1 (R Foundation for Statistical Computing, Vienna, Austria). Continuous variables were expressed as mean ± standard deviation and compared using independent *t*-tests or Mann–Whitney *U* tests as appropriate. Categorical variables were presented as frequencies and percentages and analyzed using chi-square or Fisher’s exact tests. The significance level was set at *P* < .05 (2-tailed).

## 3. Results

### 3.1. Patient characteristics

Between November 2022 and December 2023, a total of 187 patients were recruited, 92 of whom were assigned to the ERAS group and 95 to the control group. The median age was 38 years (interquartile range, 26–52 years), and the mean body mass index was 22.5 ± 3.2 kg/m². Of the total cohort, 24 patients (12.8%) reported smoking history, and 51 (27.3%) reported alcohol consumption. Regarding parity, 70 patients (37.4%) were nulliparous, 92 (49.2%) had 1 child, and 25 (13.4%) had 2 children. Baseline characteristics were comparable between the 2 groups (Table 1).

**Table 1:** Baseline demographic and clinical characteristics of study participants.

Characteristics	Overall	Control (N = 95)	ERAS (N = 92)	*P* value
Age, median (IQR)	38 (34–43)	39 (35–43)	37 (33–42)	**.009**
BMI, mean ± SD	22.463 ± 1.143	22.588 ± 1.1568	22.333 ± 1.1201	.126
Smoking history, n (%)				.933
No	163 (87.2%)	83 (44.4%)	80 (42.8%)	
Yes	24 (12.8%)	12 (6.4%)	12 (6.4%)	
Drinking history, n (%)				.310
No	136 (72.7%)	66 (35.3%)	70 (37.4%)	
Yes	51 (27.3%)	29 (15.5%)	22 (11.8%)	
Surgical history, n (%)				.972
No	138 (73.8%)	70 (37.4%)	68 (36.4%)	
Yes	49 (26.2%)	25 (13.4%)	24 (12.8%)	
Parity, n (%)				.126
0	70 (37.4%)	31 (16.6%)	39 (20.9%)	
1	92 (49.2%)	47 (25.1%)	45 (24.1%)	
2	25 (13.4%)	17 (9.1%)	8 (4.3%)	
Diagnosis, n (%)				**<.001**
Uterine fibroids	71 (38%)	57 (30.5%)	14 (7.5%)	
Ectopic pregnancy	28 (15%)	4 (2.1%)	24 (12.8%)	
Ovarian cyst	59 (31.6%)	27 (14.4%)	32 (17.1%)	
Hydrosalpinx	24 (12.8%)	2 (1.1%)	22 (11.8%)	
Abnormal uterine bleeding	5 (2.7%)	5 (2.7%)	0 (0%)	

Bold values indicate statistical significance at *P* value <.05. BMI = body mass index, ERAS = enhanced recovery after surgery, IQR = interquartile range.

### 3.2. Perioperative outcomes

Mechanical bowel preparation was performed in 48 patients (50.8%) in the control group, whereas it was omitted in all ERAS patients (*P* < .001). Postoperative gastrointestinal response patterns differed significantly between groups, with 47.1% of control patients showing delayed recovery compared to none in the ERAS group (*P* < .001). The ERAS group demonstrated significantly shorter times to first flatus (15.5 ± 1.7 vs 21.2 ± 3.5 hours; *P* < .001) and first bowel movement. Additionally, length of hospital stay was significantly reduced in the ERAS group (4.9 ± 0.9 vs 6.3 ± 1.2 days; *P* < .001; Table 2).

**Table 2:** Comparison of perioperative outcomes between ERAS and control groups.

Characteristics	Overall	Control (N = 95)	ERAS (N = 92)	*P* value
Enema, n (%)				**<.001**
Yes	95 (50.8%)	95 (50.8%)	0 (0%)	
No	92 (49.2%)	0 (0%)	92 (49.2%)	
Gastrointestinal reactions, n (%)				.279
0	176 (94.1%)	88 (47.1%)	88 (47.1%)	
1	10 (5.3%)	7 (3.7%)	3 (1.6%)	
2	1 (0.5%)	0 (0%)	1 (0.5%)	
Flatus, mean ± SD	11.155 ± 6.4067	16.821 ± 3.4301	5.3043 ± 1.9028	<.001
Defecation, mean ± SD	26.155 ± 11.321	34.989 ± 6.0274	17.033 ± 7.6711	<.001
Abdominal distension, n (%)				.555
No	171 (91.4%)	88 (47.1%)	83 (44.4%)	
Yes	16 (8.6%)	7 (3.7%)	9 (4.8%)	
Intestinal obstruction, n (%)				1.000
No	186 (99.5%)	94 (50.3%)	92 (49.2%)	
Yes	1 (0.5%)	1 (0.5%)	0 (0%)	
Duration of hospitalization, mean ± SD	5.615 ± 1.2874	6.2947 ± 1.2452	4.913 ± 0.89752	<.001
Urinary catheter, mean ± SD	18.439 ± 3.9879	21.242 ± 3.5271	15.543 ± 1.7251	<.001

Bold values indicate statistical significance at *P* value <.05. ERAS = enhanced recovery after surgery.

### 3.3. Metabolic and stress response

**Figure 1.**
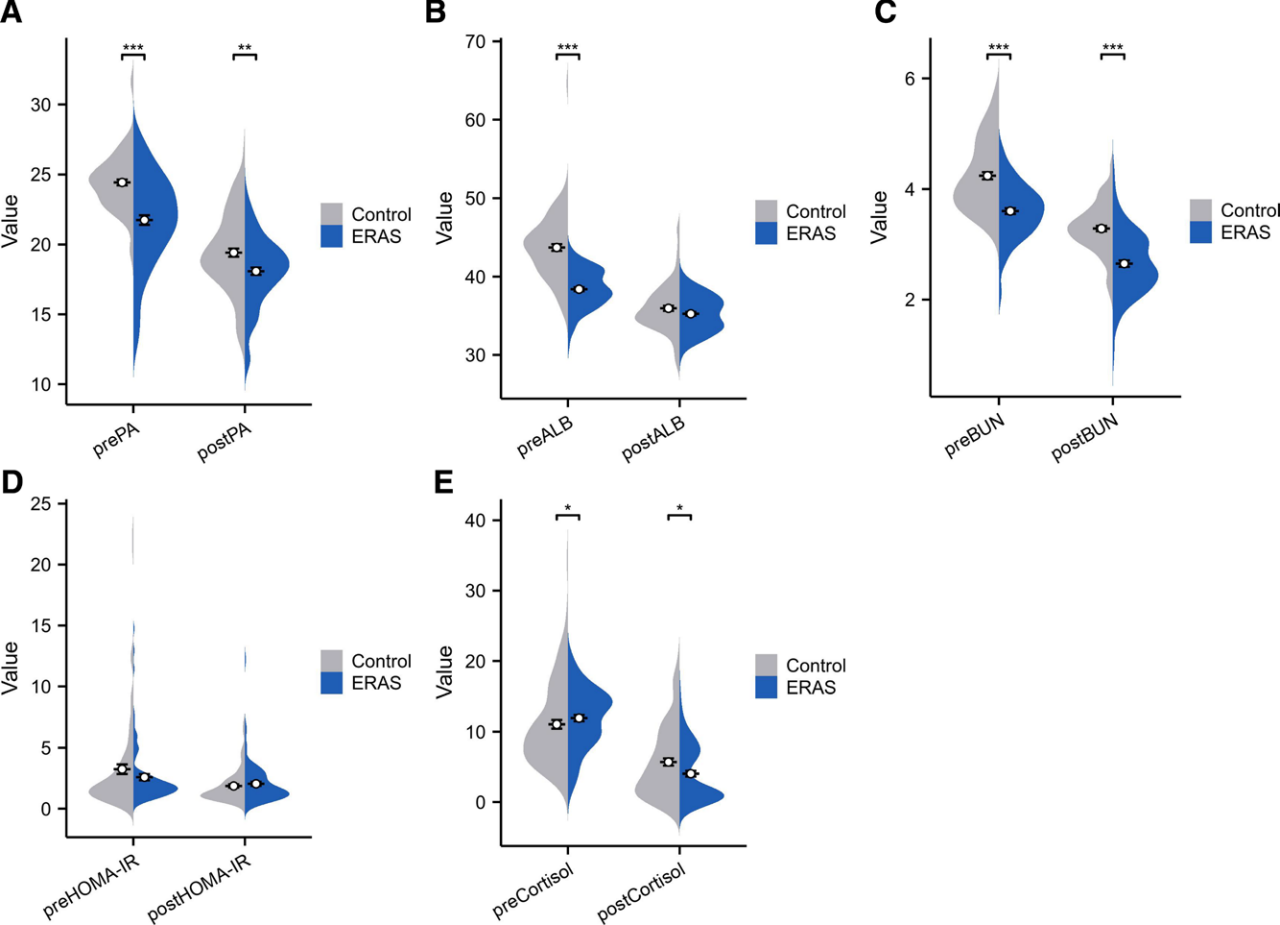
Comparison of metabolic and stress response parameters between ERAS and control groups. (A) Comparison of preoperative and postoperative prealbumin levels between groups; (B) comparison of preoperative and postoperative albumin levels between groups; (C) comparison of preoperative and postoperative blood urea nitrogen levels between groups; (D) comparison of preoperative and postoperative serum cortisol levels between groups; (E) comparison of preoperative and postoperative HOMA-IR levels between groups. Data are presented as mean ± SD, *P < .05, **P < .01, ***P < .001. ERAS = enhanced recovery after surgery, HOMA-IR = homeostatic model assessment of insulin resistance.

Preoperative nutritional markers, including prealbumin, albumin, and blood urea nitrogen, were significantly higher in the ERAS group compared to controls (all *P* < .001). Postoperative albumin levels were comparable between groups (*P* = .084). Regarding stress response, postoperative serum cortisol levels were significantly lower in the ERAS group (*P* = .012), while HOMA-IR values showed no significant differences between groups at either time point (*P* > .05; Table 3, Fig. 1).

**Table 3:** Metabolic and stress response parameters before and after surgery.

Characteristics	Overall	Control (N = 95)	ERAS (N = 92)	*P* value
Pre-PA, median (IQR)	23.6 (21.58–25.03)	24.53 (23.13–25.505)	21.87 (19.925–23.902)	**<.001**
Post-PA, median (IQR)	18.762 ± 2.7512	19.23 (17.905–21.2)	18.325 (16.925–19.67)	**.002**
Pre-ALB, median (IQR)	40.83 (37.995–43.985)	43.98 (41.12–45.695)	38.28 (36.765–40.553)	**<.001**
Post-ALB, median (IQR)	35.32 (33.715–37.295)	35.32 (34.175–37.535)	35.325 (33.492–36.938)	.156
Pre-BUN, median (IQR)	3.82 (3.5–4.32)	4.1 (3.705–4.75)	3.655 (3.29–3.94)	**<.001**
Post-BUN, mean ± sd	2.9739 ± 0.59722	3.2857 ± 0.45488	2.652 ± 0.55589	**<.001**
Pre-cortisol, median (IQR)	10.88 (7.38–14.915)	9.68 (6.38–14.975)	12.8 (9.0225–14.9)	**.040**
Post-cortisol, median (IQR)	3.67 (0.8–7.895)	4.7 (0.9–8.755)	1.275 (0.8–7.225)	**.044**
Pre-HOMA-IR, median (IQR)	1.8151 (1.3101–2.9676)	1.8493 (1.1744–3.614)	1.8151 (1.3508–2.657)	.820
Post-HOMA-IR, median (IQR)	1.4815 (0.97357–2.4669)	1.4655 (0.94876–2.3438)	1.5411 (1.0861–2.7455)	.330

Bold values indicate statistical significance at *P* value <.05. ALB = albumin, BUN = blood urea nitrogen, ERAS = enhanced recovery after surgery, HOMA-IR = homeostatic model assessment of insulin resistance, IQR = interquartile range, PA = prealbumin.

### 3.4. Healthcare costs and patient satisfaction

**Figure 2.**
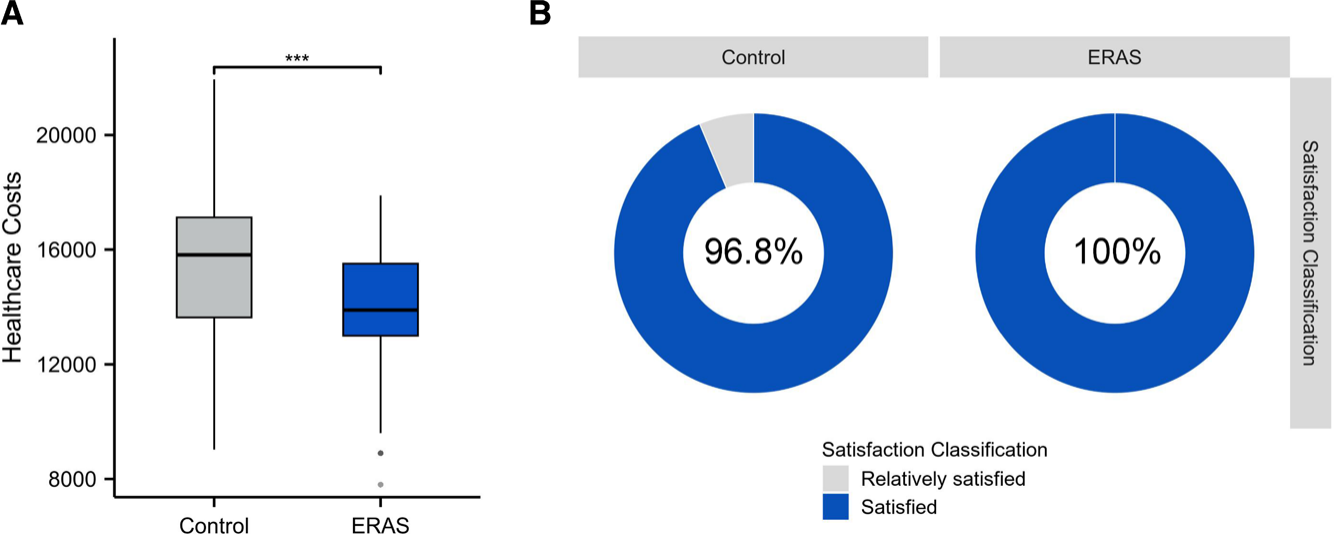
Comparison of medical costs and patient satisfaction between ERAS and control groups. (A) Comparison of total medical costs between groups. Data are presented as mean ± SD (CNY: Yuan), ***P < .001; (B) comparison of patient satisfaction at 30-day follow-up. ERAS = enhanced recovery after surgery.

Mean total healthcare costs were significantly lower in the ERAS group (13,960 ± 1967 yuan vs 15,270 ± 2856 yuan; *P* < .001). At 30-day follow-up, patient satisfaction was significantly higher in the ERAS group, with all 92 patients (100%) reporting satisfaction, compared to 89 patients (93.7%) reporting satisfaction and 6 (6.3%) reporting fair satisfaction in the control group (*P* = .042; Table 4, Fig. 2).

**Table 4:** Healthcare costs and patient satisfaction outcomes.

Characteristics	Overall	Control (N = 95)	ERAS (N = 92)	*P* value
Costs, mean ± SD	1.462e+04 ± 2538.6	1.527e+04 ± 2856	1.396e+04 ± 1966.6	<.001
Satisfaction classification, n (%)				**.042**
Satisfied	181 (96.8%)	89 (47.6%)	92 (49.2%)	
Relatively satisfied	6 (3.2%)	6 (3.2%)	0 (0%)	

Bold values indicate statistical significance at *P* value <.05. ERAS = enhanced recovery after surgery.

## 4. Discussion

This study investigates the application of the ERAS protocol in the perioperative management of patients undergoing laparoscopic gynecological surgery. The results indicate that the ERAS group significantly outperformed the control group in gastrointestinal responses, gas passage, and bowel movement times, while also demonstrating reduced urinary catheter retention time and length of stay. Additionally, the ERAS group showed better nutritional and stress indicators, lower treatment costs, and higher patient satisfaction. These findings highlight the importance of ERAS in optimizing perioperative management for laparoscopic gynecological surgery, providing solid evidence for future clinical practice.

ERAS represents a multidisciplinary perioperative optimization strategy that integrates evidence-based interventions to accelerate patient recovery. First described by Henrik Kehlet in the 1990s for colorectal surgery, it has since been adapted across surgical specialties, including orthopedics, urology, gynecology, and thoracic surgery.^[1,10,11]^ Key components include preoperative nutritional optimization, precise anesthesia, minimally invasive techniques, early catheter removal, multimodal analgesia, and individualized early rehabilitation protocols.^[6,12]^ Our findings align with this framework, demonstrating that ERAS reduces hospital length of stay, accelerates gastrointestinal function recovery, minimizes postoperative complications, and improves patient-reported outcomes.

The improved outcomes observed likely result from several complementary mechanisms. Traditional preoperative preparations often require extended fasting, which can lead to lipid accumulation in skeletal muscle, interfering with insulin signaling.^[13]^ Physiological stress during surgery releases stress hormones (such as catecholamines, cortisol, and glucagon), increasing blood glucose levels.^[14,15]^ Additionally, inflammatory responses triggered by surgery release pro-inflammatory cytokines (such as interleukin-6), further affecting insulin action.^[16]^ Changes in metabolic status and certain anesthetic agents and postoperative medications may also reduce insulin sensitivity, impacting postoperative recovery.^[17]^ The ERAS protocol shortens preoperative fasting preparation times, allowing patients to consume light meals up to 6 hours before surgery and carbohydrate-rich drinks up to 2 hours prior, thereby improving recovery.^[18,19]^ This study found that the ERAS group completely avoided gastrointestinal reactions, significantly advancing bowel motility, gas passage, and bowel movement times, demonstrating the positive impact of ERAS on bowel function recovery during the perioperative period. The significant reduction in urinary catheter retention time and length of stay not only alleviated physical discomfort, such as the risk of urinary tract infections from prolonged catheterization but also reduced patients’ exposure to the hospital environment, lowering the risk of nosocomial infections and relieving psychological stress, thereby accelerating their return to normal life. A retrospective study with propensity score matching evaluated ERAS effects on health-related quality of life (HRQL) after hysterectomy for endometrial cancer. ERAS shortened hospital stay (3 vs 5 days, *P* = .02) and improved HRQL scores at 4 weeks in physical functioning (91.6 vs 73.3, *P* < .001), role physical (81.2 vs 58.3, *P* = .02), and social functioning (58.3 vs 37.5, *P* = .01) domains, with comparable complication rates.^[20]^

Fujikuni et al found that the implementation of ERAS in gastrectomy was safe and effective, with comparable discharge rates to conventional groups within 12 days and no significant postoperative complications, along with improvements in HOMA-IR and no adverse effects on HRQL.^[21]^ This protocol aids in optimizing postoperative recovery. Our study found that the ERAS group outperformed the control group in several preoperative nutritional indicators, indicating that the preoperative preparation under this protocol may help patients maintain good nutritional status, laying a solid foundation for surgical tolerance and postoperative recovery. The reduction in postoperative cortisol levels underscores the effectiveness of the ERAS protocol in alleviating physiological stress responses, which is crucial for reducing postoperative complications and promoting tissue healing. Although some stress-related indicators, such as pre-HOMA-IR and post-HOMA-IR, showed no significant differences between the 2 groups, the overall trend still favored the ERAS group.

A randomized controlled trial by Ferrari et al found that accelerated recovery protocols significantly shortened hospital stays for gynecological surgery patients, reduced postoperative complication rates, and increased patient satisfaction.^[22]^ A study on postoperative pain predictors after laparoscopic adnexectomy found fascia closure and pneumoperitoneum duration correlated with pain, while intra-abdominal pressure and adhesiolysis did not correlate. It highlights shorter operation time and optimized suture techniques as key to minimally invasive pain management, emphasizing multifactorial pain origins and individualized interventions.^[23]^ Our study produced similar results regarding healthcare costs and patient satisfaction. The significant reduction in treatment costs for the ERAS group did not come at the expense of quality of care but was accompanied by higher patient satisfaction. This finding challenges the traditional notion that high-quality healthcare services must entail high costs, providing new insights into the rational allocation of medical resources. The lower costs may stem from ERAS measures, such as reducing unnecessary bowel preparations and shortening hospital stays, which avoid wasting healthcare resources. The tangible experiences of patients during the rapid recovery process translate into higher satisfaction ratings, further confirming the potential of ERAS to enhance the value of healthcare services.

However, this study has certain limitations. Despite efforts to ensure sample representativeness, the sample size was relatively small, and larger multicenter studies are needed to further validate the generalizability of the results. Additionally, this study primarily focused on short-term perioperative outcomes, with limited exploration of long-term prognosis following ERAS application (e.g., long-term impacts on reproductive function and incidence of chronic pain). Future research should extend follow-up periods and broaden observational indicators to comprehensively assess the overall benefits of ERAS.

## 5. Conclusion

In conclusion, this study strongly confirms the significant advantages of the ERAS protocol in the perioperative management of laparoscopic gynecological surgery, providing valuable references for clinical practice. Ongoing in-depth research is essential to drive the precise and widespread application of the ERAS concept in the gynecological field, with the potential to bring greater benefits to female patients.

## Author contributions

**Conceptualization:** Rouzi Nuermanguli, Hou Yu.

**Data curation:** Rouzi Nuermanguli, Deng Jing, Yan JiangYing, Hou Yu.

**Formal analysis:** Deng Jing, Yan JiangYing.

**Funding acquisition:** Rouzi Nuermanguli.

**Investigation:** Rouzi Nuermanguli.

**Methodology:** Rouzi Nuermanguli, Deng Jing, Hou Yu.

**Visualization:** Deng Jing, Yan JiangYing.

**Validation:** Yan JiangYing.

**Writing – original draft:** Rouzi Nuermanguli, Hou Yu.

**Writing – review & editing:** Rouzi Nuermanguli, Hou Yu.
